# Transitions in Health Insurance During the Perinatal Period Among Patients With Continuous Insurance Coverage

**DOI:** 10.1001/jamanetworkopen.2022.39803

**Published:** 2022-11-02

**Authors:** Chanup Jeung, Laura B. Attanasio, Kimberley H. Geissler

**Affiliations:** 1 University of Massachusetts Amherst School of Public Health and Health Sciences, Amherst

## Abstract

**Importance:**

Although health insurance continuity is important during the perinatal period to improve birth outcomes and reduce maternal morbidity and mortality, insurance disruptions are common. However, little is known about insurance transitions among insurance types for individuals who remained insured during the perinatal period.

**Objective:**

To examine insurance transitions for birthing individuals with continuous insurance, including those with Medicaid and Medicaid managed care coverage, before, during, and after pregnancy.

**Design, Setting, and Participants:**

This cohort study used January 1, 2014 to December 31, 2018 data from the Massachusetts All-Payer Claims Database. The sample included deliveries from January 1, 2015, to December 31, 2017, to birthing individuals aged 18 to 44 years old with continuous insurance for 12 months before and after delivery. Data were analyzed from November 9, 2021, to September 2, 2022.

**Exposure:**

Insurance type at delivery.

**Main Outcomes and Measures:**

The primary outcome was a binary indicator of any transition in insurance type from 12 months before and/or after delivery. The secondary outcomes were measures of any predelivery transition (12 months before delivery month) and any transition during the postpartum period (delivery month to 12 months post partum). Multivariate logit regression models were used to analyze the association of an insurance transition in the perinatal period with insurance type in the delivery month, controlling for age and socioeconomic status based on a 5-digit zip code.

**Results:**

The analytic sample included 97 335 deliveries (mean [SD] maternal age at delivery, 30.4 [5.5] years). Of these deliveries, 23.4% (22 794) were insured by Medicaid and 28.1% (27 347) by Medicaid managed care in the delivery month. A total of 37.1% of the sample (36 127) had at least 1 insurance transition during the 12 months before and/or after delivery. In regression-adjusted analyses, those individuals covered by Medicaid and Medicaid managed care at delivery were 47.0 (95% CI, 46.3-47.7) percentage points and 50.1 (95% CI, 49.4-50.8) percentage points, respectively, more likely to have an insurance transition than those covered by private insurance. Those covered by Marketplace plans at delivery had a 33.1% (95% CI, 31.4%-34.8%) regression-adjusted predicted probability of having a postpartum insurance transition.

**Conclusions and Relevance:**

Results of this study showed that insurance transitions during the perinatal period occurred for more than 1 in 3 birthing individuals with continuous insurance and were more common among those with Medicaid or Medicaid managed care at delivery. Further research is needed to examine the role of insurance transitions in health care use and outcomes during the perinatal period.

## Key Points

**Question:**

How frequently do insurance transitions occur among individuals with continuous insurance in the perinatal period, and what types of insurance transitions are most common?

**Findings:**

In this cohort study of 97 335 deliveries, more than 1 in 3 birthing individuals with continuous insurance experienced an insurance transition in the 12 months before and/or after giving birth. Transitions involving moving in and out of Medicaid and Medicaid managed care coverage were most common.

**Meaning:**

Findings of this study suggest that, given that insurance transitions are common in the perinatal period for people with continuous insurance, further research should examine the role of insurance transitions in perinatal care use and outcomes.

## Teaser

This cohort study assesses the frequency and timing of changes in insurance status and insurance type among individuals 12 months before and/or after delivery.

## Introduction

Health insurance is an important factor in health care access, allowing for timely diagnosis, monitoring, and treatment of health conditions.^1^ Insurance disruptions (defined as changes in insurance status, type, or plan) can lead to coverage gaps, financial distress, and administrative burden.^2,3,4^ In the US, complex insurance eligibility requirements that are based on income, family size, marital status, employment status, and pregnancy status^5^ may make birthing individuals particularly vulnerable to insurance disruptions.

The Affordable Care Act substantially increased health insurance coverage for individuals of reproductive age through Medicaid expansion and Marketplace subsidies.^6,7,8,9^ Despite these improvements, challenges remain for ensuring insurance continuity and health care access during pregnancy and post partum. Pregnancy is not a qualifying event for special enrollment in Marketplace plans outside the annual open-enrollment period in most states, but losing pregnancy-related coverage and giving birth are qualifying events.^10^ Although federal law requires Medicaid to provide coverage for pregnancy-related medical services, Medicaid eligibility for pregnant individuals can end as soon as 60 days post partum. After this period, new parents are subject to re-enrollment in their state’s regular Medicaid program, which has a much lower income threshold, particularly in states that have not expanded Medicaid.^5,10^ Marketplace subsidies are available at income levels that overlap with pregnancy-related Medicaid and thus may be an option before and after pregnancy for those with an income between 138% and 400% of the federal poverty level.

Access to care is important throughout the perinatal period.^11,12^ Although health insurance coverage gaps are particularly detrimental to health care access, switching health insurance plans disrupts continuity of care even for those who remain insured. Different insurance benefits and provider networks may necessitate a change in health care practitioners or lead to a reduction in access.^13,14^ Insurance transitions within and among private and Marketplace insurance plans may reset deductibles or cost-sharing. Previous studies of perinatal insurance transitions have had study populations that included individuals with continuous coverage,^5,11,15^ but health insurance changes among these individuals have not been characterized in detail. Understanding changes in health insurance types among this population may be particularly important given that Medicaid expansion has reduced uninsurance spells during the perinatal period, partly through increasing transitions between Medicaid and private insurance.^15^ Limited research has categorized insurance type beyond private coverage, Medicaid coverage, and uninsurance^1,5,16^ or by including insurance status over more granular periods and across a longer interval post partum.^17,18^ One study examined Marketplace coverage during the perinatal period and found that only one-third of individuals with Marketplace coverage had it continuously from conception through post partum,^17^ underscoring the importance of understanding coverage patterns by specific insurance types. We are not aware of any studies examining perinatal transitions between Medicaid and Medicaid managed care despite evidence that provider networks, including for obstetric care, vary substantially across and within these Medicaid plan types.^19,20^

To better understand the frequency and types of health insurance transitions, we examined insurance transitions for birthing individuals with continuous insurance before, during, and after pregnancy. We identified transitions at each stage of the perinatal period, categorized these transitions by type of insurance transition, and examined factors associated with an insurance transition. We hypothesized that birthing individuals insured by Medicaid or Medicaid managed care in the delivery month were more likely to have an insurance transition than those with other insurance types.

## Methods

This retrospective cohort study was approved by the University of Massachusetts Amherst Institutional Review Board, which waived the informed consent requirement due to the use of preexisting secondary data. We followed the Strengthening the Reporting of Observational Studies in Epidemiology (STROBE) reporting guideline.^21^

### Data and Analytic Sample

The Massachusetts All-Payer Claims Database (APCD) collects medical claims records and insurance enrollment information from Medicaid, Medicaid managed care plans, and most private insurers within the state.^22,23^ Using *International Classification of Diseases, Ninth Revision* and *International Statistical Classification of Diseases and Related Health Problems, Tenth Revision* diagnosis and procedure codes,^24^ we identified individuals with a live birth between January 1, 2015, and December 31, 2017. We restricted the sample to births to individuals aged 18 to 44 years at delivery who resided in Massachusetts for 12 months before and after delivery. We further limited the sample to individuals who were continuously insured in primary medical plan by any reporting insurer for 12 months before and after the delivery month.

### Measures

The primary outcome was a binary measure of any insurance transition during the 12 months before and/or after delivery. We defined an insurance transition as a change in insurance type in adjacent months. Using eligibility data, we categorized coverage for each month into 5 mutually exclusive types: private, Medicaid, Medicaid managed care, Marketplace, and Health Safety Net. For secondary outcomes, we constructed measures of any predelivery transition (12 months before delivery month) and any transition during the postpartum period (delivery month to 12 months post partum).

In Massachusetts during this period, people enrolling in Medicaid could generally choose between primary care clinician plans administered by the state Medicaid program or plans administered by managed care organizations. Those who opted for plans administered by the state Medicaid program (Medicaid) can switch to a managed care plan (Medicaid managed care) anytime, whereas those who selected plans administered by managed care organizations can switch only during annual plan selection periods. Enrollees can also change between Medicaid plan types based on Medicaid redeterminations or moving between regions.

The Health Safety Net in Massachusetts is a program that pays for certain services at acute care hospitals and community health centers for residents without insurance or who are underinsured; it does not qualify as minimum essential coverage.^25^ We included individuals with the Health Safety Net in the sample with continuous insurance because they had access to some care and limited Medicaid coverage for the delivery hospitalization.^26^ Under a section 1115 waiver, the Massachusetts Medicaid program began a demonstration project in which they implemented accountable care organizations (ACOs) starting in March 2018.^27^ Most people covered by a Medicaid ACO have an ACO plan administered by a Medicaid managed care insurer.^28^ We coded managed care–administered Medicaid ACOs as Medicaid managed care and did not consider individuals enrolled in Medicaid managed care before implementation as having an insurance transition. To examine the association of insurance transition with socioeconomic status, we identified whether an individual lived in a 5-digit zip code with a median household income in the lowest quartile^29^ or a 5-digit zip code with concentrated poverty^30^ (defined as areas with an overall poverty rate greater than 30%).^31^

### Statistical Analysis

We calculated descriptive statistics, with statistical comparisons between those with and without an insurance transition using χ^2^ tests. Then, we constructed a longitudinal analysis of insurance type, showing transitions across types using a Sankey diagram for the full sample and for those insured by a Marketplace plan at any point.

To account for patient characteristics in describing the probability of insurance transitions according to insurance type at delivery, we estimated multivariate logistic regressions with 3 binary outcomes: any transition during the study period (25 months), any predelivery transition, and any postpartum transition. The primary independent variable in each regression was a categorical measure of insurance type in the delivery month. We also controlled for age at delivery, patient residence in a zip code in the lowest quartile of median income, and patient residence in a zip code with concentrated poverty. We estimated predicted probabilities of insurance transitions averaged across observed covariates. Race and ethnicity data were not available in the Massachusetts APCD.

We used 2-tailed statistical tests, with *P* < .05 defined as the level of statistical significance and used SAS, version 9.4 (SAS Institute Inc) and Stata/MP, version 16.1 (Stata Corp LLC) to perform the analysis. Data were analyzed from November 9, 2021, to September 2, 2022.

We conducted 2 sensitivity analyses. First, we repeated the main analysis with the sample restricted to individuals with continuous insurance from a reporting insurer during different time windows (2, 3, and 6 months before and after delivery). In this sensitivity analysis, if someone switched from a reporting private insurer to unobserved, we included this as an insurance transition. With nearly complete coverage of this population,^32^ the person was most likely to be transitioning to a nonreporting private plan from a self-insured employer,^23^ but classifying this as a transition for individuals with private insurance was a conservative calculation of the differences between insurance types. Second, we restricted the sample to those who had deliveries before March 1, 2017, and repeated the analysis, which avoided confounding issues from the Medicaid program’s transition to the ACO model in March 2018.

## Results

The analytic sample included 97 335 deliveries to birthing individuals with a mean (SD) age at delivery of 30.4 (5.5) years (eFigure 1 in the Supplement). Of these deliveries, 23.4% were covered by Medicaid and 28.1% by Medicaid managed care (Table). More than one-third (37.1%) of deliveries had an insurance transition during the 12 months before and/or after delivery. Among those with any insurance transition, 24.0% had postpartum insurance transitions only, 38.8% had predelivery transitions only, and 37.2% had both predelivery and postpartum transitions. About one-third (33.5%) of the sample resided in a zip code in the lowest quartile of median income, and 5.8% resided in a zip code with concentrated poverty. Those with private insurance in the delivery month were less likely to have an insurance transition than those with other insurance types.

**Table.** Sample Characteristics (N = 97 335)

Characteristic	Overall, No. (%)	Insurance transition 12 mo before or after delivery		
		No. (%)		*P* value^a^
		No (n = 61 208)	Yes (n = 36 127)	
Outcomes				
Any transition	36 127 (37.1)	0	36 127 (37.1)	NA
Predelivery transition only	14 012 (14.4)	0	14 012 (14.4)	
Postpartum transition only	8662 (8.9)	0	8662 (8.9)	
Predelivery and postpartum transitions	13 453 (13.8)	0	13 453 (13.8)	
No. of transitions				
0	61 208 (62.9)	61 208 (62.9)	0	NA
1	12 181 (12.5)	0	12 181 (12.5)	
2	12 732 (13.1)	0	12 732 (13.1)	
≥3	11 214 (11.5)	0	11 214 (11.5)	
Age group, y				
18-24	15 908 (16.6)	7262 (7.5)	8646 (8.9)	<.001
25-34	58 100 (56.0)	36 866 (37.9)	21 234 (21.8)	
35-44	23 327 (23.4)	17 080 (17.5)	6247 (6.4)	
Delivery year				
2015	34 362 (35.3)	22 814 (23.4)	11 548 (11.9)	<.001
2016	31 417 (32.3)	19 917 (20.5)	11 500 (11.8)	
2017	31 556 (32.4)	18 477 (19.0)	13 079 (13.4)	
Insurance type at delivery				
Private	43 056 (44.2)	38 814 (39.9)	4242 (4.4)	<.001
Medicaid	22 794 (23.4)	9898 (10.2)	12 896 (13.2)	
Medicaid managed care	27 347 (28.1)	11 040 (11.3)	16 307 (16.8)	
Marketplace	3009 (3.1)	1360 (1.4)	1649 (1.7)	
Health Safety Net	1129 (1.2)	96 (0.1)	1033 (1.1)	
Delivery mode				
Vaginal	65 481 (67.3)	41 191 (42.3)	24 290 (25.0)	.03
Cesarean	31 854 (32.7)	20 017 (20.6)	11 837 (12.2)	
Patient residence in zip code with lowest-quartile median income	32 635 (33.5)	16 698 (17.2)	15 937 (16.4)	<.001
Patient residence in zip code with concentrated poverty	5657 (5.8)	3014 (3.1)	2643 (2.7)	<.001

Abbreviation: NA, not applicable.

^a^ *P* values were calculated using χ^2^ for binary and categorical variables.

**Figure 1.**
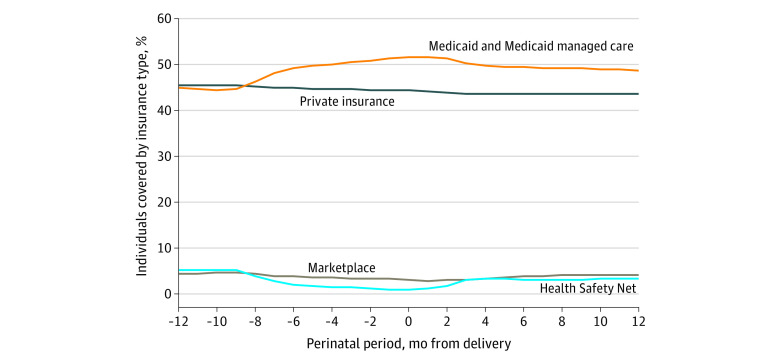
Compositions of Insurance Types by Months Before and After Delivery

Figure 1 shows the proportion of individuals covered by each insurance type over the perinatal period. The most prevalent insurance type was private, consistently covering approximately 44.3% of the sample. In the predelivery period, the proportion of individuals with any type of Medicaid coverage increased from 44.5% to 51.5%. Three months after delivery, the proportion of individuals with the Health Safety Net increased rapidly from 1.2% to 3.1%. Marketplace plans covered between 3.1% and 4.5% of individuals in the sample.

**Figure 2.**
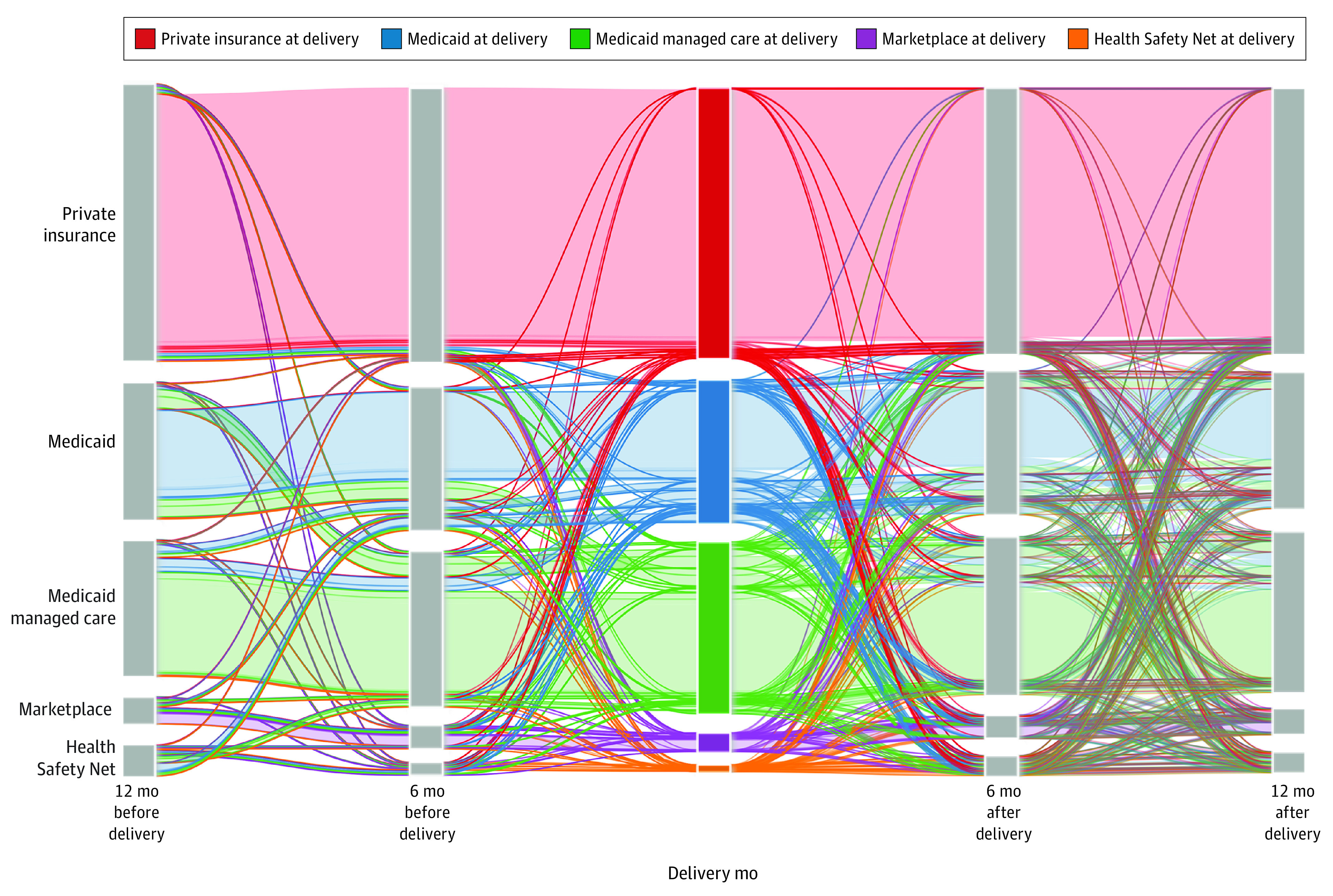
Insurance Transitions Over the Study Period

A longitudinal analysis of insurance type over the perinatal period (Figure 2) found that, although those with private insurance had relatively stable coverage with the same insurance type during the perinatal period, individuals with the other insurance types had more frequent and complex transitions across insurance types. After restricting the sample to those with Marketplace plans at any time point (eFigure 4 in the Supplement), we found that the proportion of individuals with Marketplace plans was higher 12 months before (4.5%) and 12 months after (4.3%) delivery than in the delivery month (3.1%). Those covered by Marketplace plans at delivery had a 54.6% (95% CI, 52.8%-56.4%) regression-adjusted predicted probability of having any transition and a 33.1% (95% CI, 31.4%-34.8%) regression-adjusted predicted probability of having a postpartum insurance transition. Two months post partum, 94.6% of individuals remained with the same insurance type, and this proportion decreased to 86.6% (eFigure 2 in the Supplement). By 12 months post partum, 81.5% remained with the same insurance type. Meanwhile, 35.6% of individuals with the Health Safety Net in the delivery month changed their insurance, mostly to any Medicaid plan (80.1% of those with transition) (eFigure 3 in the Supplement).

Six months after delivery, more than 96.3% of individuals with private insurance in the delivery month remained enrolled in private insurance. Moreover, 80.1% of individuals with Medicaid and 78.7% of individuals with Medicaid managed care had the same insurance type. Among those who transitioned from Medicaid, 64.3% switched to Medicare managed care. Among those who changed their insurance from Medicaid managed care, 69.4% switched to Medicaid. Individuals covered by Marketplace plans mostly retained this insurance type (81.0%) 6 months after delivery, with similar proportions switching to private (7.4%), any Medicaid (6.2%), or the Health Safety Net (5.5%).

By 12 months post partum, the overall proportion of individuals who retained their insurance type decreased from 6 months post partum (from 86.6% to 81.5%), but the pattern of insurance transitions was similar. Those with private insurance in the delivery month had the highest retention rate (94.8%), and a small proportion of these individuals switched to any Medicaid (2.4%). Retention rates decreased to 70.9% for Medicaid and 73.6% for Medicaid managed care, and insurance transitions for individuals with any Medicaid type occurred mostly between Medicaid and Medicaid managed care. Most people with Marketplace plans in the delivery month remained covered by a Marketplace plan (72.6%), with many more switching to private insurance (13.5%) than at 6 months post partum. Among those with the Health Safety Net in the delivery month, 62.7% had a different insurance type by 12 months after delivery.

**Figure 3.**
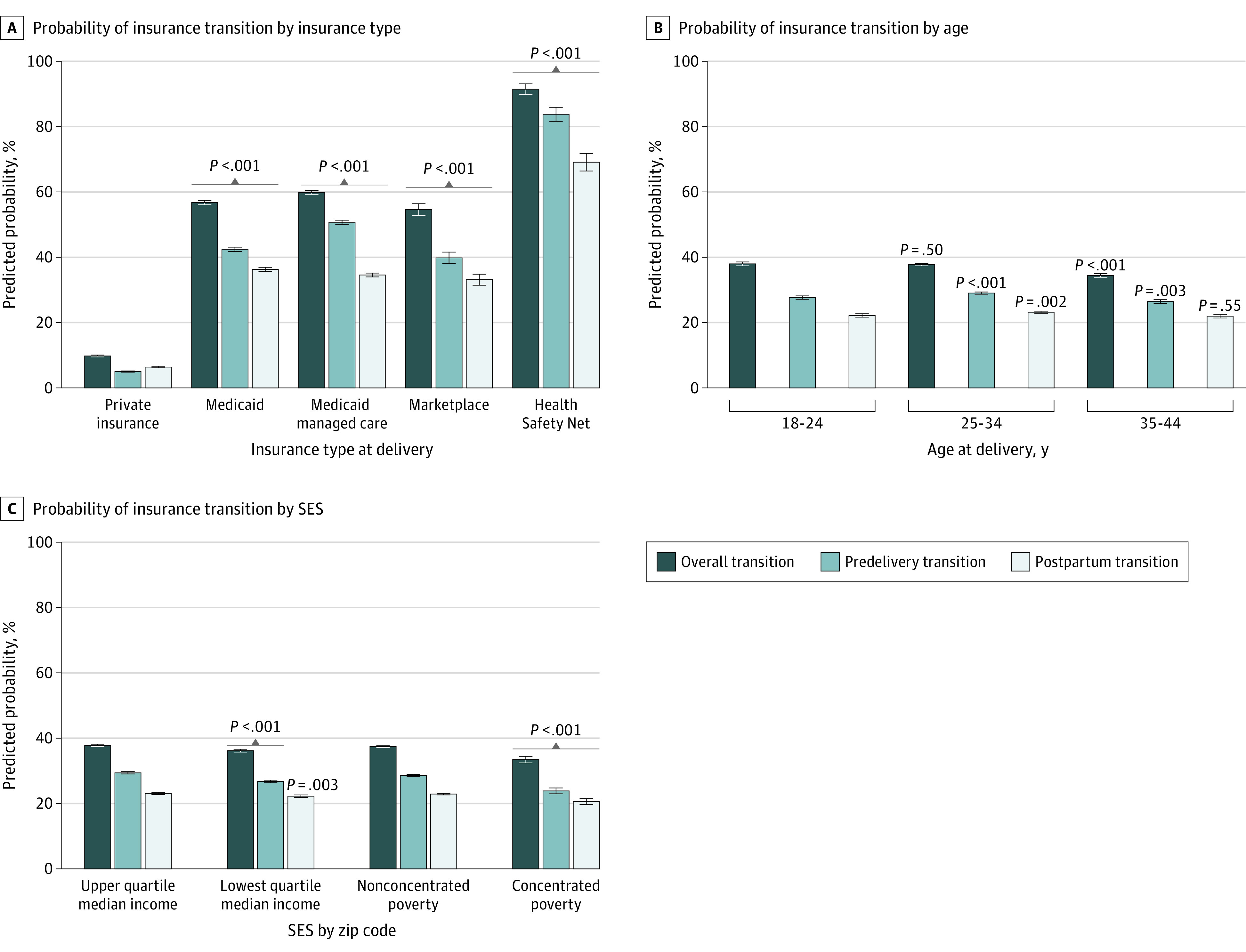
Regression-Adjusted Predicted Probability of Insurance Transitions Predicted probabilities were calculated using logistic regressions controlling for insurance type at delivery, age, and socioeconomic status (SES) based on zip code. Error bars present 95% CIs. *P* values indicate that each outcome (overall, postpartum transition, and predelivery transition) is statistically significantly different from the reference category (private insurance, age 18-24 years, residence in a zip code with upper-quartile median income, and residence in a zip code without concentrated poverty).

Figure 3 shows the predicted probabilities of insurance transitions according to logistic regressions, with an insurance transition as the outcome. The pattern and magnitude of predicted probabilities were similar across periods (overall, predelivery transition, and postpartum transition). After accounting for other factors, the predicted probability of an insurance transition was considerably higher for people covered by Medicaid or Medicaid managed care than for those with private insurance in the delivery month. The likelihood of having an insurance transition was higher for individuals with Medicaid (47.0 [95% CI, 46.3-47.7] percentage points more likely) and those with Medicaid managed care (50.1 [95% CI, 49.4-50.8] percentage points more likely) in the delivery month than for those with private insurance. These differences narrowed in the postpartum period to 29.9 (95% CI, 29.2-30.6) percentage points for individuals with Medicaid and 28.2 (95% CI, 27.5- 28.8) percentage points for those with Medicaid managed care.

Individuals aged 35 to 44 years were 3.5 (95% CI, −4.4 to −2.7) percentage points less likely to have an insurance transition than adults aged 18 to 24 years. Individuals living in zip codes with lowest-quartile median income were 1.6 (95% CI, −2.2 to −1.0) percentage points less likely to have an insurance transition than those living in zip codes with upper-quartile median income. Those residing in zip codes with concentrated poverty were 4.0 (95% CI, −5.0 to −2.9) percentage points less likely to have an insurance transition than those living in other areas.

The sensitivity analyses (eTables 1-3 in the Supplement) revealed that the relative magnitude and significance of odds ratios (ORs) for insurance type at delivery were similar across the time windows (2, 3, and 6 months before and after delivery). For example, with the 12 months before and after delivery time window, the OR of any insurance transitions was the highest for those with the Health Safety Net (OR, 99.90; 95% CI, 80.82-123.49), followed by Medicaid managed care (OR, 13.85; 95% CI, 13.27-14.45), Medicaid (OR, 12.19; 95% CI, 11.67-12.74), and Marketplace (OR, 11.17; 95% CI, 10.32-12.08) (eTable 1 in the Supplement). The rank of OR by insurance type was consistent across the time windows, and this pattern was the same for predelivery transition (eTable 2 in the Supplement) and postpartum transition (eTable 3 in the Supplement). After restricting the sample to those with deliveries before March 2017 (eTable 4 in the Supplement), we found that the predicted probabilities were similar to those in the main analysis.

## Discussion

We investigated insurance transitions among birthing individuals with continuous insurance and found more than one-third had at least 1 transition during the year before and the year after delivery. Although previous research on perinatal insurance transitions characterized transitions between private insurance, Medicaid, and uninsurance,^5,6,10,12,33^ to our knowledge, the current study was the first to examine specific timing and types of insurance transitions during the perinatal period among individuals with continuous insurance and to follow these individuals across the entirety of the postpartum year. The use of high-quality, comprehensive administrative data in this study allowed us to observe month-to-month insurance transitions across insurance types at a more granular level.

Previous literature has mostly examined perinatal insurance transitions up to 6 months post partum.^5,12,33^ Results of this study showed that extending the time frame of consideration provided greater insights into the insurance transitions within the postpartum year, which is increasingly recognized to be critical for maternal health in the short and long terms.^34^ Insurance transitions increased substantially over the postpartum year, with 5.4% of people having a different insurance type at 2 months after delivery, 13.4% having a different type at 6 months after delivery, and 18.5% having a different type at 12 months post partum compared with the insurance type in the delivery month.

Health insurance continuity is critical to maintaining health care access during the perinatal period.^11,12,15^ The mean age of first birth is increasing, and the prevalence of chronic conditions, such as obesity, hypertension, and diabetes, has substantially increased among young women.^35,36^ Growing pregnancy-related risks highlight the importance of coordinated care among primary, obstetric, and specialty care.^37,38^ However, frequent insurance transitions could threaten health outcomes, particularly for birthing individuals with chronic conditions and/or high-risk pregnancy who particularly benefit from care in the postpartum year.^39,40,41^ Unlike previous studies, this study focused on birthing individuals who continuously had health insurance and examined specific types of transitions. As a result, the sample may more accurately characterize the population with insurance coverage in states with expanded Medicaid and postpartum Medicaid extensions, wherein uninsurance in the perinatal period will become much more uncommon. As of May 2022, extended Medicaid coverage for a full year post partum has been implemented or is planned to be implemented in 31 states.^42^

The most prevalent type of insurance transition was switching between Medicaid and Medicaid managed care. These transitions, although potentially not as disruptive as switching between or among private and Marketplace plans with potential changes in deductibles and/or copayment structures in addition to changes in the provider network, may still have implications for access to care. Provider networks have been shown to vary substantially across Medicaid managed care plans,^20^ specifically for obstetric practitioners.^19^ Although less frequent, we found that perinatal insurance transitions were also common among individuals with private insurance.

Approximately 8% of birthing individuals in this sample have a Marketplace plan at any point, making it an important coverage source. With a regression-adjusted predicted probability of experiencing any transition of 54.6% and with both predelivery and postpartum transitions being common for those with Marketplace coverage in the delivery month, understanding coverage stability for this population is important. Previous work found that a small percentage of working-age adults transition between Marketplace and Medicaid plans each year.^43^ In the perinatal period specifically, the results of this study are similar to those in a recent study, which reported that transitions were common for individuals with Marketplace coverage.^17^ In the Marketplace sample, transitions to and from Medicaid and Medicaid managed care were most common.

### Limitations

This study had some limitations. First, we used data from Massachusetts, which has the lowest uninsurance rate in the US.^32^ However, the substantial progress made toward providing insurance to birthing individuals during and after pregnancy suggests that insurance coverage for this population in Massachusetts is similar to that in many other states. Massachusetts has similar income eligibility limits for pregnant people compared with the national median^44^ and is 1 of 39 states with Medicaid expansion,^45^ 1 of 31 states that have implemented or are planning a 1-year postpartum extension of coverage,^42^ and 1 of 25 states that waive the 5-year waiting period for pregnant lawfully residing immigrants.^10^ Second, the Massachusetts APCD does not include characteristics associated with insurance eligibility that may affect the probability and type of insurance transitions, including income,^18^ workforce participation, race and ethnicity,^46^ and marital status; further research may examine the role of these factors for those with continuous insurance. Third, the Massachusetts APCD, similar to other state APCDs after the Supreme Court decision in *Gobeille v Liberty Mutual Insurance Co*.,^47^ does not include full reporting by private insurers due to selective reporting by self-insured employers.^48^ The analytic sample was disproportionately composed of individuals with Medicaid coverage compared with the full set of births in Massachusetts from 2016 to 2017.^49^ In addition, because we did not observe people with continuous insurance from nonreporting insurance plans, the results may overstate the transition rates in private insurance during the perinatal period, which would bias downward the estimates of the differences between private insurance and other insurance types.

## Conclusions

This cohort study found that insurance transitions throughout the perinatal period were common, with more than 1 in 3 birthing people switching their coverage during this period. Switching across Medicaid insurance types was the most prevalent type of transition; thus, better understanding of these transitions is important as states extend postpartum Medicaid coverage and expand the use of managed care in Medicaid programs. Future research is needed to investigate the implications of insurance transitions within and between coverage types for health care use and outcomes.

## Notes

Supplement. **eFigure 1.** Sample Restriction Criteria **eFigure 2.** Percentage of Deliveries with Same Insurance Type vs. Different Insurance Type at 2, 6, and 12 Months Postpartum **eFigure 3.** Postpartum Insurance Changes **eFigure 4.** Insurance Transitions for Individuals Insured by a Marketplace Plan at Any Time Point **eTable 1.** Full Regression Results for Sensitivity Analysis Examining Sample Restriction for Months of Continuous Insurance and Observation Required (Any Transition) **eTable 2.** Sensitivity Analysis Examining Sample Restriction for Months of Continuous Insurance and Observation Required (Pre-Delivery Transition) **eTable 3.** Sensitivity Analysis Examining Sample Restriction for Months of Continuous Insurance and Observation Required (Postpartum Transition) **eTable 4.** Predicted Probabilities for Sensitivity Analysis to Sample Limitation of Deliveries Before March 2017
