# Error in the Results Section

**DOI:** 10.1001/jamanetworkopen.2022.48174

**Published:** 2022-12-06

In the Original Investigation titled “Transitions in Health Insurance During the Perinatal Period Among Patients With Continuous Insurance Coverage” published online November 2, 2022, there was an error in the Results section regarding the name of the insurance coverage to which individuals with Medicaid transitioned. The sentence should state “Among individuals who transitioned from Medicaid, 64.3% switched to Medicaid managed care.” The article has been corrected.^1^
