# Supplementary material for: Transitions in Health Insurance During the Perinatal Period Among Patients With Continuous Insurance Coverage
Source: JAMA Netw Open. 2022 Nov 2;5(11):e2239803. doi: 10.1001/jamanetworkopen.2022.39803 (PMC9631105; doi:10.1001/jamanetworkopen.2022.39803)
Supplement: Supplement. — eFigure 1. Sample Restriction Criteria eFigure 2. Percentage of Deliveries with Same Insurance Type vs. Different Insurance Type at 2, 6, and 12 Months Postpartum eFigure 3. Postpartum Insurance Changes eFigure 4. Insurance Transitions for Individuals Insured by a Marketplace Plan at Any Time Point eTable 1. Full Regression Results for Sensitivity Analysis Examining Sample Restriction for Months of Continuous Insurance and Observation Required (Any Transition) eTable 2. Sensitivity Analysis Examining Sample Restriction for Months of Continuous Insurance and Observation Required (Pre-Delivery Transition) eTable 3. Sensitivity Analysis Examining Sample Restriction for Months of Continuous Insurance and Observation Required (Postpartum Transition) eTable 4. Predicted Probabilities for Sensitivity Analysis to Sample Limitation of Deliveries Before March 2017 [file jamanetwopen-e2239803-s001.pdf]

Supplemental Online Content

Jeung C, Attanasio LB, Geissler KH. Transitions in health insurance during the perinatal period among patients with continuous insurance coverage. *JAMA Netw Open*. 2022;5(11):e2239803. doi:10.1001/jamanetworkopen.2022.39803

eFigure 1. Sample Restriction Criteria

eFigure 2. Percentage of Deliveries with Same Insurance Type vs. Different Insurance Type at 2, 6, and 12 Months Postpartum

eFigure 3. Postpartum Insurance Changes

eFigure 4. Insurance Transitions for Individuals Insured by a Marketplace Plan at Any Time Point

eTable 1. Full Regression Results for Sensitivity Analysis Examining Sample Restriction for Months of Continuous Insurance and Observation Required (Any Transition)

eTable 2. Sensitivity Analysis Examining Sample Restriction for Months of Continuous Insurance and Observation Required (Pre-Delivery Transition)

eTable 3. Sensitivity Analysis Examining Sample Restriction for Months of Continuous Insurance and Observation Required (Postpartum Transition)

eTable 4. Predicted Probabilities for Sensitivity Analysis to Sample Limitation of Deliveries Before March 2017

This supplemental material has been provided by the authors to give readers additional information about their work.

© 2022 Jeung C et al. *JAMA Network Open.*

eFigure 1. Sample Restriction Criteria

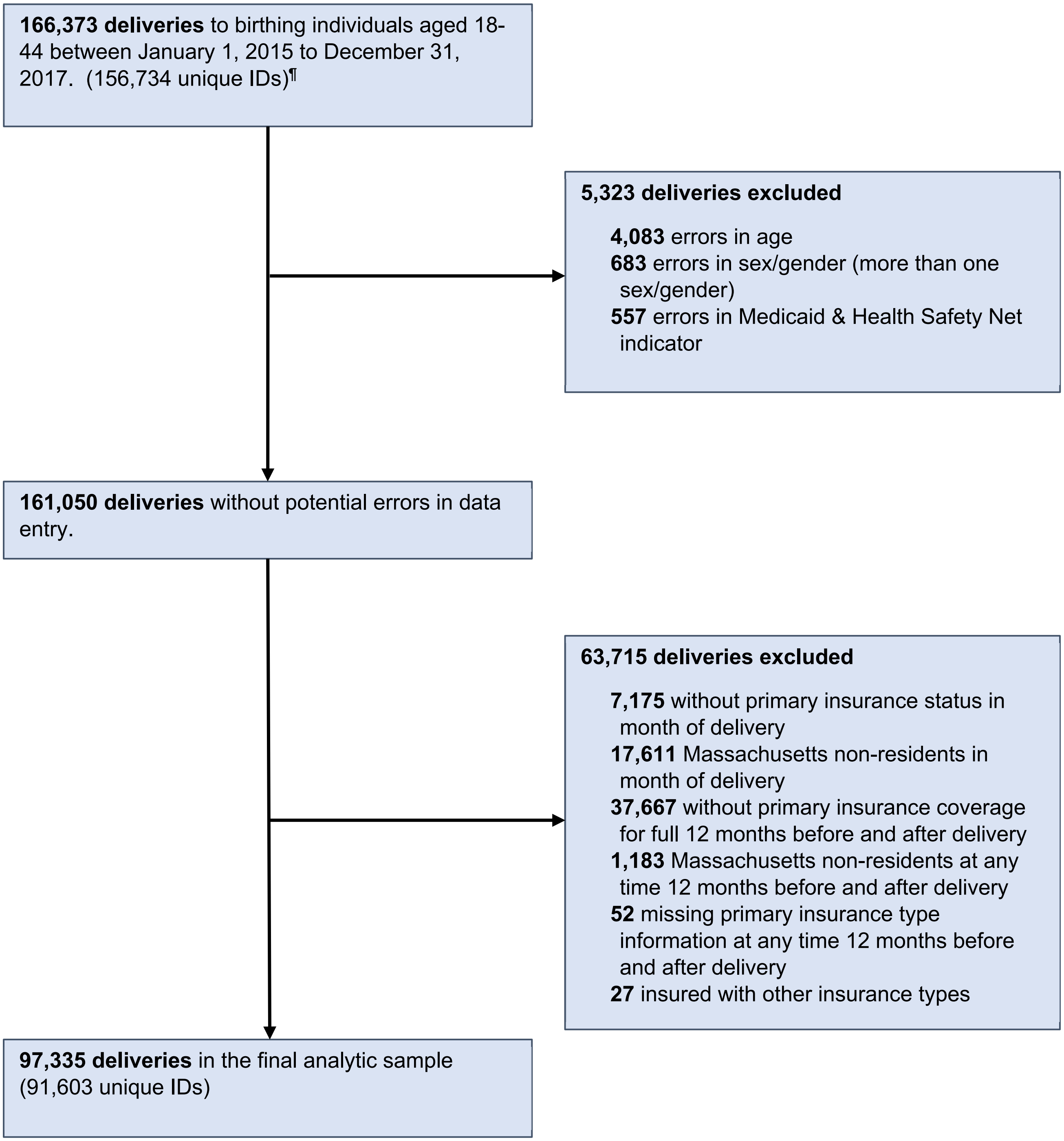

¶ Note: Births within 180 days of a prior birth were excluded from the sample.

© 2022 Jeung C et al. *JAMA Network Open.*

eFigure 2. Percentage of Deliveries with Same Insurance Type vs. Different Insurance Type at 2, 6, and 12 Months Postpartum

Panel A: 2 months Postpartum

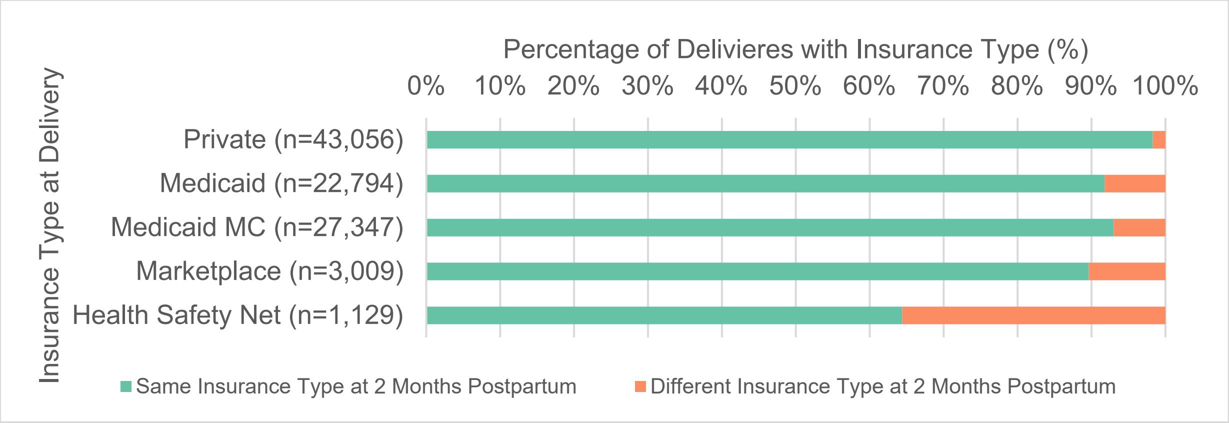

Panel B: 6 months Postpartum

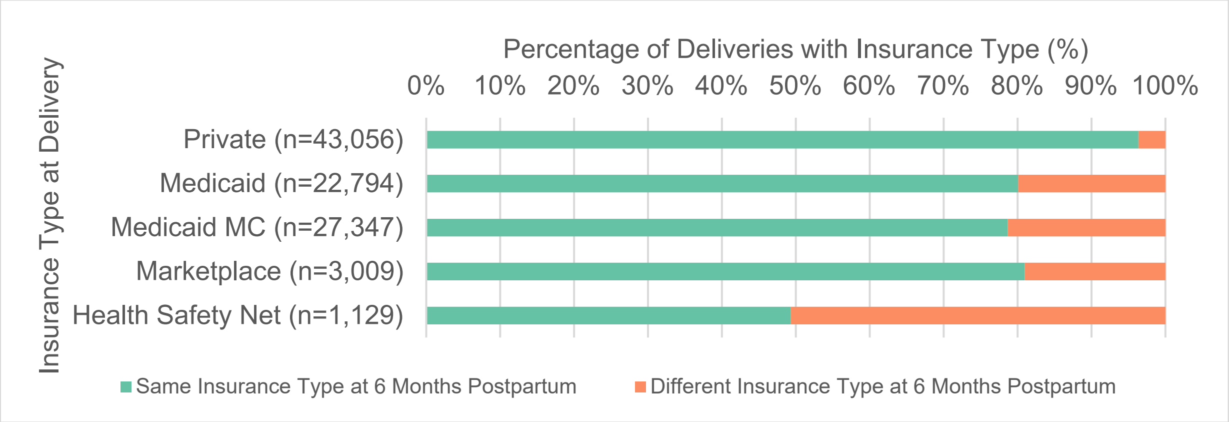

Panel C: 12 months Postpartum

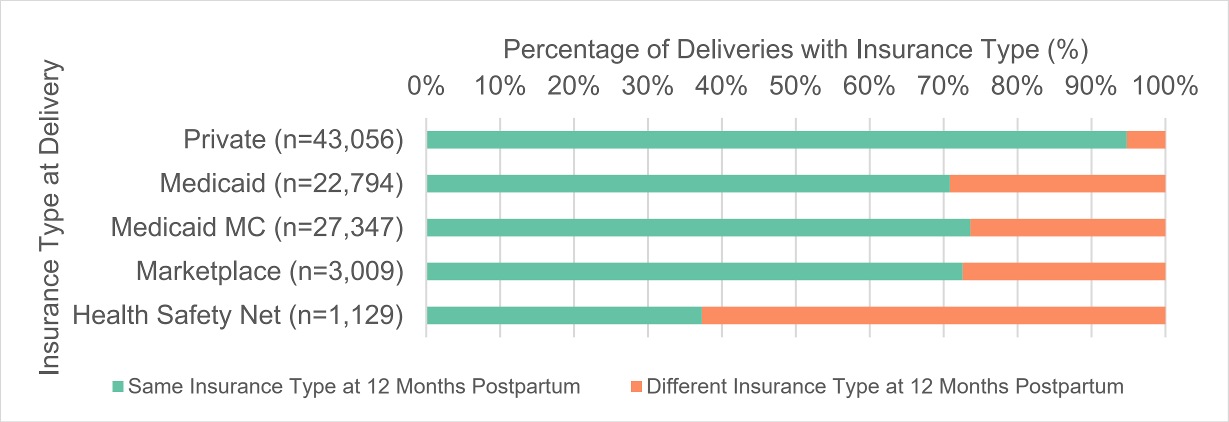

Note: n=97,335. Insurance status 2 months, 6 months, and 12 months postpartum were compared with the insurance type in the delivery month.

© 2022 Jeung C et al. *JAMA Network Open.*

eFigure 3. Postpartum Insurance Changes

Panel A: 2 months Postpartum

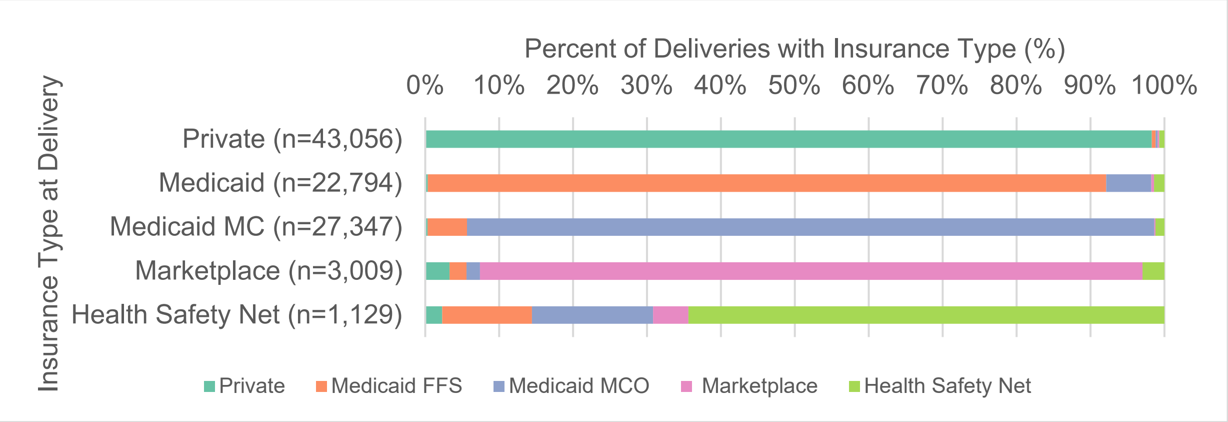

Panel B: 6 months Postpartum

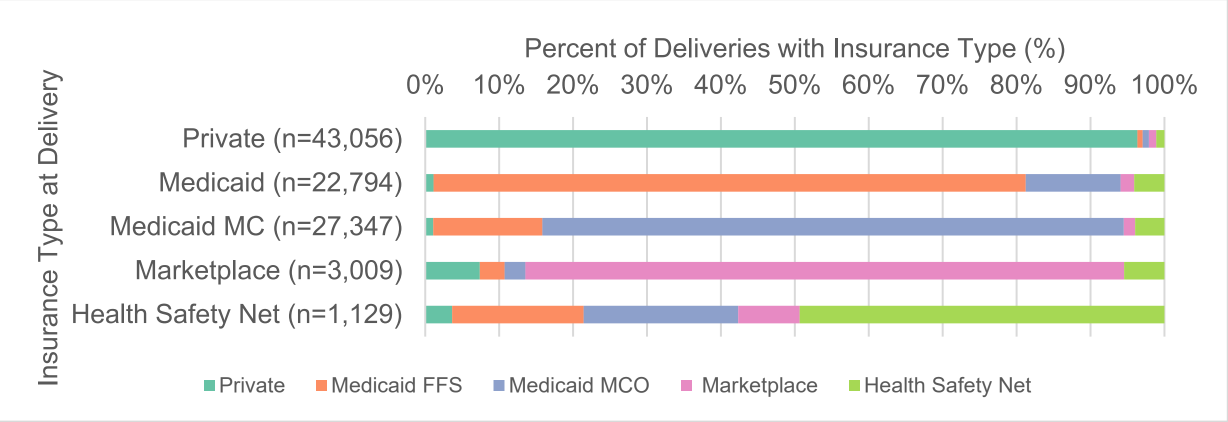

Panel C: 12 months Postpartum

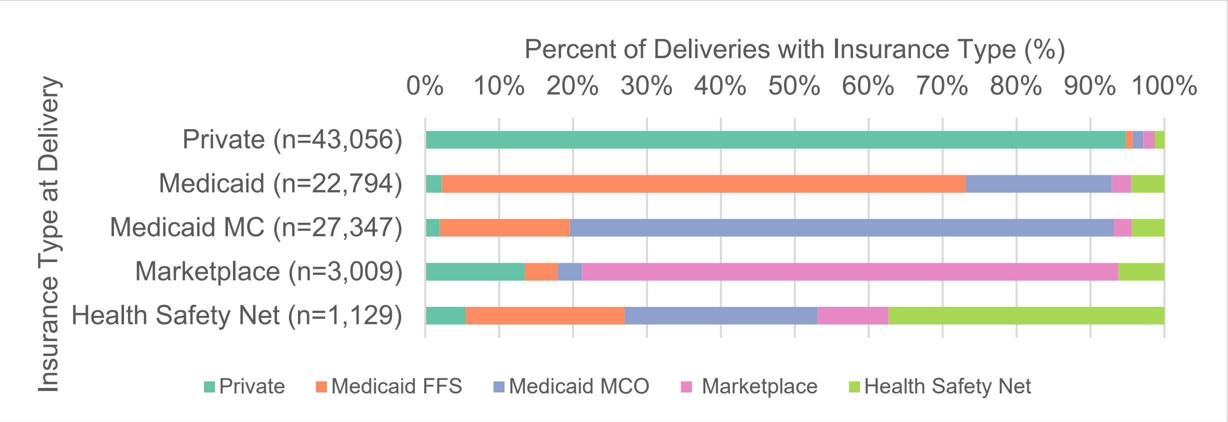

Note: n=97,335. Insurance status 2 months, 6 months, and 12 months postpartum were compared with the insurance status in delivery month.

© 2022 Jeung C et al. *JAMA Network Open.*

eFigure 4. Insurance Transitions for Individuals Insured by a Marketplace Plan at Any Time Point

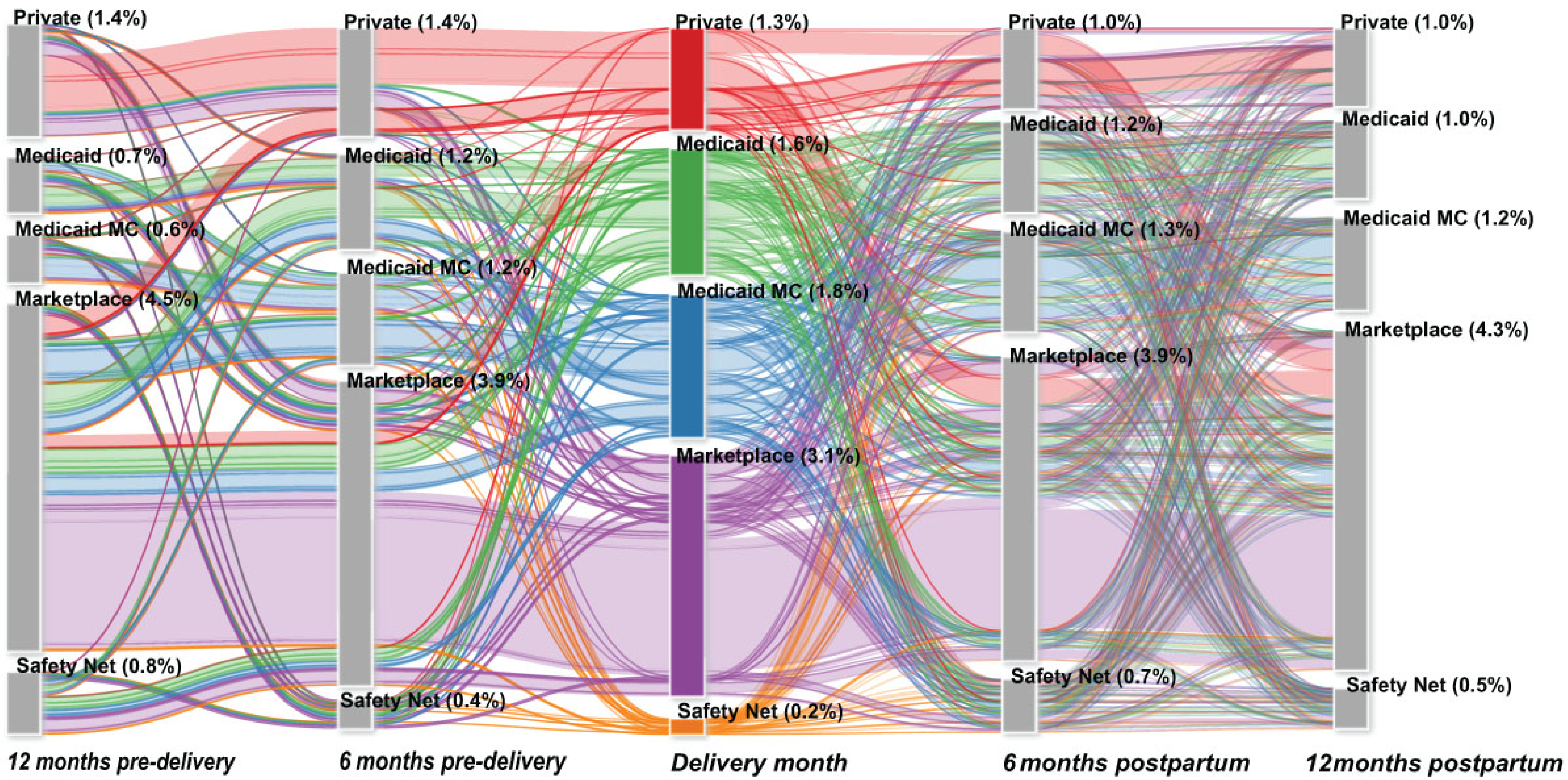

Note: n=7,847 deliveries with Marketplace insurance at any time point. Sample is restricted to 18-44 years old women who had deliveries between January 2015 – December 2017, and further restricted to those who had Marketplace insurance at any time point. Numbers shown in the diagram are % of the full analytic sample (n=97,335).

© 2022 Jeung C et al. *JAMA Network Open.*

eTable 1. Full Regression Results for Sensitivity Analysis Examining Sample Restriction for Months of Continuous Insurance and Observation Required (Any Transition)

Outcome: Any Transition during 12 month pre-delivery to 12 month postpartum period	±12 Months Continuously Insured and Observed (Primary Analytic Sample)		±6 Months Continuously Insured and Observed		±3 Months Continuously Insured and Observed		±2 Months Continuously Insured and Observed	
	OR	*95% CI*	*OR*	*95% CI*	*OR*	*95% CI*	*OR*	*95% CI*
Insurance at Delivery (Ref=Private)								
Medicaid	12.19***	11.67 - 12.74	6.16***	5.95 - 6.38	5.05***	4.89 - 5.22	4.89***	4.73 - 5.05
Medicaid MC	13.85***	13.27 - 14.45	6.55***	6.33 - 6.77	5.31***	5.14 - 5.48	5.13***	4.98 - 5.30
Marketplace	11.17***	10.32 - 12.08	5.59***	5.21 - 5.99	4.59***	4.29 - 4.91	4.37***	4.08 - 4.67
Health Safety Net	99.90***	80.82 - 123.49	42.81***	34.87 - 52.56	33.67***	27.47 - 41.26	30.57***	25.04 - 37.32
Age (Ref= Age 18-24)								
Age 25-34	0.99	0.95 - 1.03	0.97	0.94 - 1.01	0.98	0.95 - 1.01	0.99	0.96 - 1.03
Age 35-44	0.82***	0.78 - 0.86	0.79***	0.76 - 0.83	0.80***	0.77 - 0.83	0.81***	0.78 - 0.84
Patient Residence in ZIP Code with Lowest Quartile Median Income	0.91***	0.88 - 0.94	0.91***	0.88 - 0.94	0.91***	0.88 - 0.93	0.90***	0.88 - 0.93
Patient Residence in ZIP Code with Concentrated Poverty	0.79***	0.75 - 0.84	0.79***	0.75 - 0.83	0.79***	0.75 - 0.84	0.79***	0.75 - 0.83
Number of Observations	97,335		115,550		125,042		129,031	

*** p<0.01, ** p<0.05

Note: Odds ratios are based on logistic regression that used any insurance transition (overall, pre-delivery, and postpartum) during 25 month study period as outcome variable. Sample is restricted to 12 months (primary analytic sample), 6 months, 3 months, and 2 months continuously insured before and after delivery from delivery month.

© 2022 Jeung C et al. *JAMA Network Open.*

eTable 2. Sensitivity Analysis Examining Sample Restriction for Months of Continuous Insurance and Observation Required (Pre-Delivery Transition)

Outcome: Any Transition during 12 month pre-delivery period	±12 Months Continuously Insured and Observed (Primary Analytic Sample)		±6 Months Continuously Insured and Observed		±3 Months Continuously Insured and Observed		±2 Months Continuously Insured and Observed	
	OR	*95% CI*	*OR*	*95% CI*	*OR*	*95% CI*	*OR*	*95% CI*
Insurance at Delivery (Ref=Private)								
Medicaid	14.08***	13.36 - 14.84	10.42***	10.01 - 10.85	9.67***	9.32 - 10.04	9.65***	9.31 - 10.01
Medicaid MC	19.69***	18.70 - 20.72	13.01***	12.51 - 13.53	11.82***	11.40 - 12.26	11.74***	11.33 - 12.16
Marketplace	12.64***	11.61 - 13.75	8.95***	8.33 - 9.61	8.07***	7.54 - 8.63	7.84***	7.33 - 8.38
Health Safety Net	99.41***	84.37 - 117.14	59.47***	50.83 - 69.57	50.70***	43.50 - 59.09	47.75***	41.05 - 55.54
Age (Ref= Age 18-24)								
Age 25-34	1.09***	1.05 - 1.14	1.05***	1.01 - 1.09	1.05***	1.01 - 1.08	1.06***	1.03 - 1.10
Age 35-44	0.93***	0.88 - 0.97	0.84***	0.80 - 0.88	0.84***	0.80 - 0.87	0.85***	0.81 - 0.88
Patient Residence in ZIP Code with Lowest Quartile Median Income	0.84***	0.81 - 0.87	0.87***	0.84 - 0.89	0.87***	0.84 - 0.90	0.87***	0.84 - 0.89
Patient Residence in ZIP Code with Concentrated Poverty	0.73***	0.69 - 0.78	0.74***	0.70 - 0.79	0.75***	0.71 - 0.79	0.74***	0.70 - 0.78
Number of Observations	97,335		115,550		125,042		129,031	

*** p<0.01, ** p<0.05

Note: Odds ratios are based on logistic regression that used any insurance transitions during 12 month pre-delivery period as outcome. Sample is restricted to 12 months, 6 months, 3 months, and 2 months continuously insured from delivery month.

© 2022 Jeung C et al. *JAMA Network Open.*

eTable 3. Sensitivity Analysis Examining Sample Restriction for Months of Continuous Insurance and Observation Required (Postpartum Transition)

Outcome: Any Transition during 12 month postpartum period	±12 Months Continuously Insured and Observed (Primary Analytic Sample)		±6 Months Continuously Insured and Observed		±3 Months Continuously Insured and Observed		±2 Months Continuously Insured and Observed	
	OR	*95% CI*	*OR*	*95% CI*	*OR*	*95% CI*	*OR*	*95% CI*
Insurance at Delivery (Ref=Private)								
Medicaid	8.35***	7.95 - 8.78	3.09***	2.98 - 3.20	2.21***	2.13 - 2.28	2.03***	1.96 - 2.10
Medicaid MC	7.74***	7.38 - 8.13	3.38***	3.26 - 3.50	2.60***	2.52 - 2.69	2.48***	2.40 - 2.55
Marketplace	7.26***	6.67 - 7.91	2.94***	2.73 - 3.16	2.28***	2.13 - 2.44	2.16***	2.02 - 2.31
Health Safety Net	32.83***	28.76 - 37.49	11.84***	10.52 - 13.32	8.74***	7.80 - 9.80	8.06***	7.20 - 9.02
Age (Ref= Age 18-24)								
Age 25-34	1.07***	1.02 - 1.11	1.10***	1.06 - 1.14	1.11***	1.07 - 1.15	1.12***	1.08 - 1.16
Age 35-44	0.98	0.93 - 1.04	1.03	0.99 - 1.08	1.03	0.99 - 1.08	1.05**	1.00 - 1.09
Patient Residence in ZIP Code with Lowest Quartile Median Income	0.95***	0.91 - 0.98	0.94***	0.91 - 0.97	0.94***	0.91 - 0.97	0.94***	0.91 - 0.97
Patient Residence in ZIP Code with Concentrated Poverty	0.86***	0.80 - 0.92	0.84***	0.79 - 0.89	0.83***	0.79 - 0.88	0.83***	0.79 - 0.88
Number of Observations	97,335		115,550		125,042		129,031	

*** p<0.01, ** p<0.05

Note: Odds ratios are based on logistic regression that used any insurance transitions during 12 month postpartum period as outcome. Sample is restricted to 12 months, 6 months, 3 months, and 2 months continuously insured from delivery month.

© 2022 Jeung C et al. *JAMA Network Open.*

eTable 4. Predicted Probabilities for Sensitivity Analysis to Sample Limitation of Deliveries Before March 2017

	Any Transition	*95% CI*	Pre-delivery Transition	*95% CI*	Postpartum Transition	*95% CI*
Insurance at Delivery						
Private	0.097***	0.093 - 0.100	0.047***	0.044 - 0.049	0.066***	0.063 - 0.068
Medicaid	0.503***	0.494 - 0.511	0.396***	0.388 - 0.405	0.293***	0.286 - 0.301
Medicaid MC	0.604***	0.597 - 0.611	0.507***	0.500 - 0.514	0.346***	0.339 - 0.353
Marketplace	0.560***	0.538 - 0.582	0.430***	0.408 - 0.451	0.324***	0.304 - 0.345
Health Safety Net	0.927***	0.910 - 0.945	0.887***	0.866 - 0.908	0.676***	0.645 - 0.707
Age						
Age 18-24	0.358***	0.351 - 0.365	0.270***	0.264 - 0.277	0.196***	0.190 - 0.202
Age 25-34	0.358***	0.354 - 0.362	0.277***	0.274 - 0.281	0.214***	0.210 - 0.218
Age 35-44	0.330***	0.324 - 0.337	0.257***	0.250 - 0.263	0.204***	0.198 - 0.210
Patient Residence in Zip Code with Upper Quartile Median Income	0.358***	0.354 - 0.362	0.282***	0.278 - 0.287	0.211***	0.208 - 0.215
Patient Residence in ZIP Code with Lowest Quartile Median Income	0.344***	0.339 - 0.349	0.259***	0.255 - 0.264	0.203***	0.199 - 0.208
Patient Residence in Zip Code with non-Poverty Concentration	0.356***	0.353 - 0.359	0.276***	0.273 - 0.279	0.210***	0.207 - 0.213
Patient Residence in ZIP Code with Concentrated Poverty	0.311***	0.300 - 0.322	0.231***	0.221 - 0.241	0.181***	0.171 - 0.191
Number of Observations	70,461		70,461		70,461	

*** p<0.01, ** p<0.05

Note: Deliveries are limited to those who had deliveries before March 2017 to avoid confounding effects from the Medicaid program's ACO transition. Predicted probabilities are comparable to Figure 3 in the main results.

© 2022 Jeung C et al. *JAMA Network Open.*
